# A facile method for generating polypyrrole microcapsules and their application in electrochemical sensing

**DOI:** 10.1007/s00604-022-05512-1

**Published:** 2022-10-08

**Authors:** Piyanut Pinyou, Vincent Blay, Jirawan Monkrathok, Pattanaphong Janphuang, Kantapat Chansaenpak, Jaruwan Pansalee, Sireerat Lisnund

**Affiliations:** 1 School of Chemistry, Institute of Science, Suranaree University of Technology, 111 University Ave., Nakhon Ratchasima, 30000 Thailand; 2 Department of Microbiology and Environmental Toxicology, University of California at Santa Cruz, Santa Cruz, CA 95064 USA; 3 Synchrotron Light Research Institute, 111 University Ave., Nakhon Ratchasima, 30000 Thailand; 4 Institute of Research and Development, Suranaree University of Technology, 111 University Ave.., Nakhon Ratchasima, 30000 Thailand; 5 National Nanotechnology Center, National Science and Technology Development Agency, Thailand Science Park, Pathum Thani, 12120 Thailand; 6 Department of Applied Chemistry, Faculty of Science and Liberal Arts, Rajamangala University of Technology Isan, 744, Suranarai Rd., Nakhon Ratchasima, 30000 Thailand

**Keywords:** Screen-printed electrodes, Encapsulation, Biosensor, Capsule immobilization, Microfluidics, Chronoamperometry

## Abstract

A facile and rapid strategy to generate polypyrrole microcapsules is reported. The strategy is compatible with a vortex mixer and with a microfluidic chip for droplet generation, allowing a > 100-fold reduction in particle size. The sub-micron particle sizes obtained can also be tuned to some extent based on the chip geometry. The capsules can be kept stably in solution and can be transferred onto electrochemical devices. As an application example, we casted the polypyrrole capsules generated onto screen-printed electrodes, leading to a significant increase in their electroactive surface area and capacitance. The electrodes were further modified with glucose dehydrogenase (GDH) to fabricate glucose biosensors. The introduction of polypyrrole microcapsules increased the dynamic range of the glucose sensor to ca. 300% compared with that of the electrode without polypyrrole microcapsules. The resulting glucose sensor is operated at a constant applied potential of 0.20 V vs. Ag/AgCl (3 M KCl) in an air-equilibrated electrolyte. At this potential, the sensor showed a linear range from 1.0 to 9.0 mM glucose with a sensitivity of 3.23 µA cm^−2^ mM^−1^ (*R*^2^ = 0.993). The limit of detection obtained was 0.09 mM, and the reproducibility was 3.6%. The method allows generating polypyrrole microcapsules without surfactants or organic solvents and may enable new opportunities in the design of biosensors, electronic devices, and molecular delivery.

**Graphical abstract:**

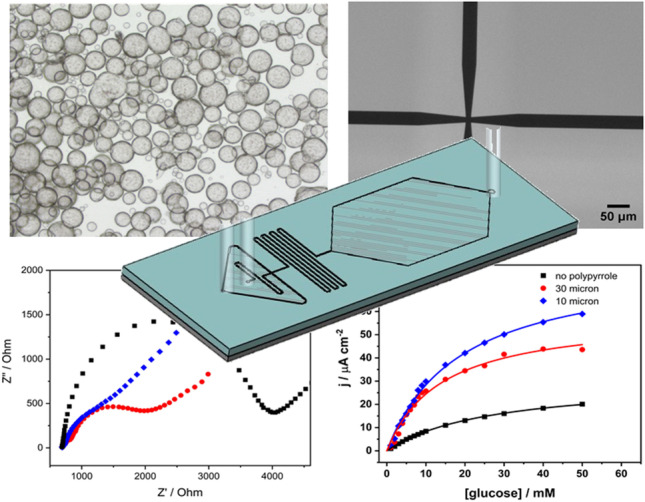

**Supplementary information:**

The online version contains supplementary material available at 10.1007/s00604-022-05512-1.

## Introduction

Polypyrrole (Ppy) was one of the first conjugated polymers synthesized. [1] Ppy offers very interesting properties such as high conductivity under physiological conditions, good chemical and thermal stability (150 °C), strong adsorptive properties toward gasses, proteins and DNA, biodegradability, and excellent biocompatibility. [2] Indeed, polypyrrole is being actively investigated to develop drug delivery devices, implants, and antibacterial coatings. [3–6] The polymerization mechanism is generally considered to involve the formation of a radical cation, which is followed by the reaction of a radical-cation with a neutral monomer or by radical-cation/radical-cation coupling to terminate the reaction. [7] The electron vacancies in the polymer backbone created during this process are charge-compensated by “dopant” anions, which can affect its electrical properties. Chloride, in particular, was reported to provide higher conductivity than other common anions. [8] More effective dopants have also been reported. [9]

A variety of methods have been proposed to initiate the polymerization of pyrrole, including photochemistry, electrochemical polymerization, and chemical oxidation with a variety of oxidizing agents. In the latter case, surfactants such as polyvinylpyrrolidone (PVP), polyvinyl alcohol (PVA), sodium dodecyl sulfate (SDS), or cetyltrimethyl ammonium (CTAB) are often used. Also, high concentrations of oxidant are generally applied. By modifying the temperature and the pH, it is possible to affect the morphology of the resulting material. [10, 11] Recently, H_2_O_2_ generated in situ by polyphenol oxidase extracts has been used to catalyze the formation of polypyrrole and later serve as the biorecognition element in an electrochemical sensor. [12] However, this mild synthesis route requires very long times for polypyrrole to develop (> 96 h) and did not demonstrate significant performance improvements over polypyrrole prepared conventionally.

The incorporation of polypyrrole particles as a strategy to modify electrodes has proved beneficial in a variety of electrochemical applications. If the particles are small enough, they can help accelerate the electron transfer in bioelectrodes. For example, Pothipor et al. demonstrated an increase in sensing performance by introducing small amounts of polypyrrole in a microRNA electrochemical sensor. [13] Thin films of polypyrrole have been used as supports for enzymes for their adsorptive properties. [14] If the load of polypyrrole is relatively high, it can increase the capacitance of the electrode, which can be useful for different electronic devices. [15–19]

In this work, we report a facile methodology for the creation of micrometric, thin-walled polypyrrole microcapsules. The preparation does not require the addition of surfactants or organic solvents and can be carried out on the benchtop. We demonstrate that the method can be implemented in microfluidic devices, enabling a > 100-fold reduction in the capsule size. As an application example, the sub-micron capsules prepared were used to modify electrodes and improve the performance of biosensors.

## Materials and methods

### Reagents

Pyrrole (reagent grade, Sigma-Aldrich), cetyltrimethylammonium chloride (CTAB, > 98%, TCI), and iron (III) chloride (FeCl_3_, reagent grade, 97%, Sigma-Aldrich) were purchased and used without further purification. Nafion perfluorinated resin solution 5 wt.% and β-nicotinamide adenine dinucleotide hydrate (NAD^+^, > 95%) were obtained from Sigma-Aldrich. Dyes used included methyl green (0.1% w/v aq. soln., Alfa Aesar), trypan blue (stain 0.4%, Gibco), and McCormick Red Food Color (a mixture of Allura Red AC and erythrosine). Toluidine blue O (TBO) and D-glucose were purchased from TCI Chemicals (Japan). Potassium ferricyanide (K_3_Fe(CN)_6_), potassium ferrocyanide trihydrate (K_4_Fe(CN)_6_.3H_2_O), and potassium chloride (KCl) were purchased from Kemaus (Australia). Type I water (Millipore Direct-Q 3R) was used to prepare aqueous solutions. The SU-8 developer used for chip fabrication was purchased from Acros Organics.

### Preparation of capsules using a vortex

A fresh stock solution of 4 M FeCl_3_ was prepared by dissolving 3.244 g FeCl_3_ in 5 mL water. This stock was then diluted to prepare the aqueous solutions. Water at neutral pH was used for the dilution, and no buffer was used. To prepare the polypyrrole dispersions, a typical total volume of 110 µL was prepared. An appropriate volume of aqueous solution was transferred to a 0.5-mL PCR tube. The appropriate amount of pyrrole was then added, and the tube was immediately vortexed on a Thermolyne Maxi Mix II at full speed (3000 rpm) for 10 s. The capsules were freshly prepared prior to the study and kept in the preparation mixture at room temperature until used. Table S1 summarizes the main formulations explored in this work.

### Imaging

Optical microscopy images were taken on a Zeiss Axio Observer Z1. A drop (ca. 15 µL) of the droplet-containing solution was placed on a glass slide and imaged in brightfield or phase contrast mode. Objectives used were 5 × , 10 × , or 20 × . The light source was typically operated at 3.3–4.0 V, so that exposure times were in the range of 20–40 ms while using 80% of the dynamic range of the color camera. When imaging in epifluorescence mode, a green filter was used. In this case, a metal halide lamp was used, and fluorescent signal was recorded on a monochromatic camera. Intensity and exposure time were adjusted to use ca. 30% of the camera dynamic range.

### Design and fabrication of microfluidic chips using X-ray lithography

**Fig. 1:**
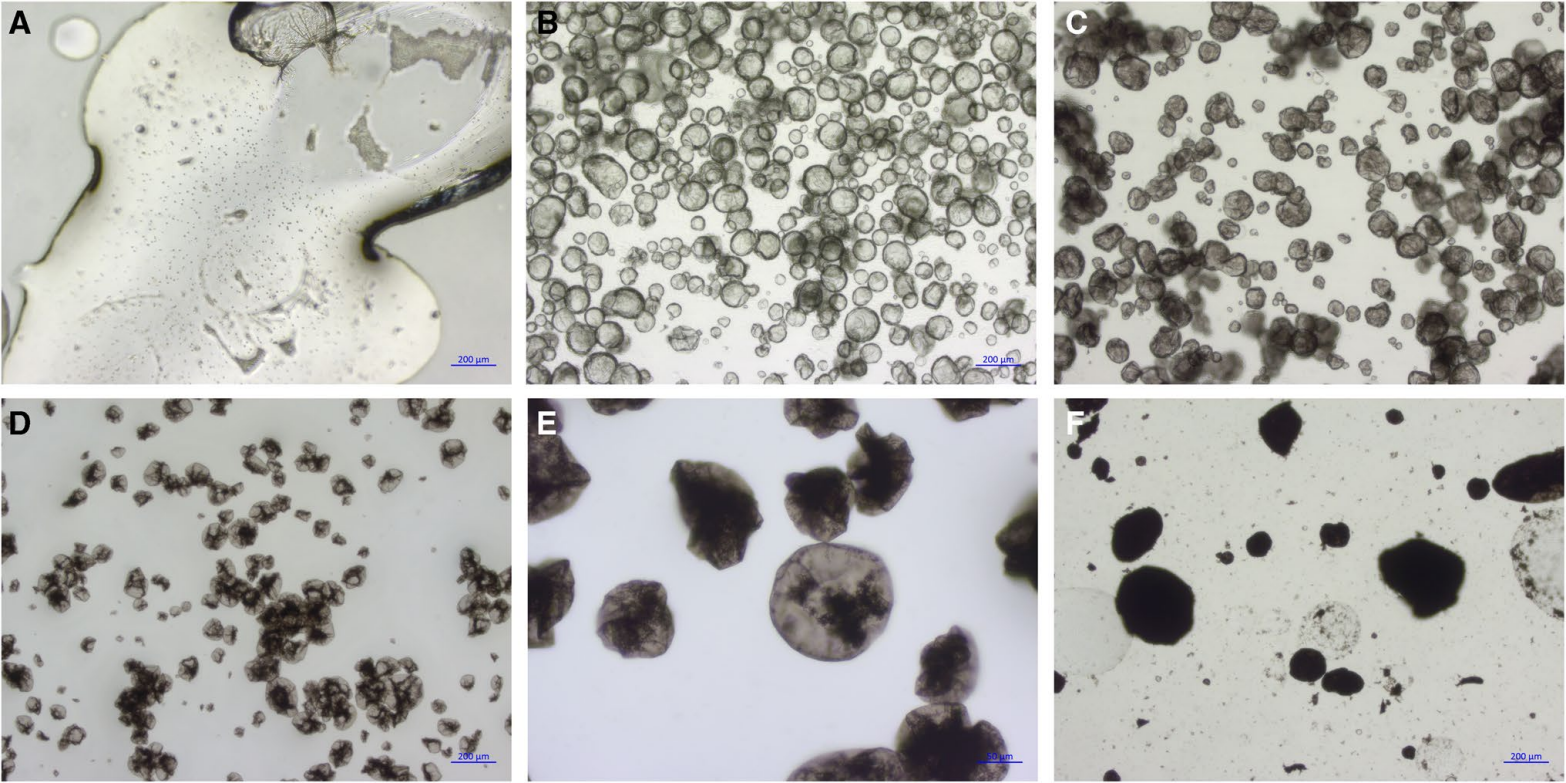
Effect of FeCl_3_ oxidant concentration in the preparation of Ppy microcapsules. **a** 0 mM, **b** 25 mM, **c** 50 mM, **d** 100 mM, **e** 0.25 M, and **g** 1.0 M. A volume ratio organic:aqueous = 10:1 was maintained in all cases

Microfluidic chips were designed to achieve the generation of droplets between the pyrrole monomer and the oxidant solution. The chip features a micropattern of channels with widths of 10 and 30 µm and a fixed height of 50 µm, as shown in Fig. 1. Different widths at the channel intersection were evaluated to affect the size of the droplets generated. Once the two input solutions are mixed, the reacting mixture circulates along a zig-zag channel, allowing polymerization to take place. The capsules then slow down in a wider collection pool, facilitating imaging before they are extracted at the other end of the pool.

Given its high aspect ratio, the device was fabricated using X-ray lithography at the Beamline 6—Deep X-ray Lithography, Synchrotron Light Research Institute (Public Organization), Thailand. First, a microchannel design was produced using CAD, and a transparent mask for photolithography was fabricated (Delta Mask B.V., The Netherlands). A 15-μm-thick AZp4620 photoresist was spin-coated on a graphite substrate, and it was soft-baked on a hot plate at 95 °C for 5 min (Figure S7a). The photomask was placed on a photoresist and exposed to a UV dose of 240 mJ cm^−2^ (Figure S7b). The pattern was then developed by solvent washing (Figure S7c). Figure S7d illustrates the forward electroplating performed at 0.5 mA cm^−2^ for 6 days, creating a thin gold layer on the graphite. Finally, AZp4620 photoresist was removed by 400 k AZ-developer—deionized water (1:4), as depicted in Figure S7e.

The next step was to transfer the designed pattern from the X-ray mask using X-ray lithography. In preparation, SU-8 photoresist was coated on a glass slide and soft-baked using a leveled hot plate, at 65 °C for 5 min, then at 95 °C for 10 min, and allowed to cool down at room temperature. This resulted in very smooth surfaces to be used as a substrate [20] as shown in Figure S8a. The X-ray mask was placed on photoresist and set up in the X-ray scanning system of the BL6, followed by an X-ray exposure of 150,000 mJ cm^−2^ as shown in Figure S8b. Afterwards, SU-8 photoresist was cross-linked in a post-exposure bake, using a leveled hot plate at 65 °C for 5 min, followed by 10 min at 95 °C, and allowed to cool down at room temperature. The pattern was developed by solvent washing, resulting in the SU-8 microchannel mold (Figure S8c).

PDMS is widely used to fabricate microfluidic chips due to its favorable optical and mechanical properties. The fabrication process of the SU-8 and PDMS is shown in Figure S9. PDMS was prepared using siloxane oligomer and curing agent at a 10:1 weight ratio, which were mixed and degassed to remove gas bubbles. PDMS was poured over the SU-8 master mold structure (Figure S9a). The cross-linking of PDMS took place on a hotplate at 70 °C for 1 h. [21] The PDMS was then peeled off from the master to obtain the replica of the microfluidic chip (Figure S9b). The PDMS was permanently bonded to a glass slide by applying oxygen plasma to the PDMS surface (200 W for 1 min), which changes the PDMS surface to a hydrophilic state. [22] The PDMS slab was then aligned with a glass slide and connected with silicone tubing as shown in Figure S9c.

### Preparation of capsules using microfluidic chips

**Fig. 2:**
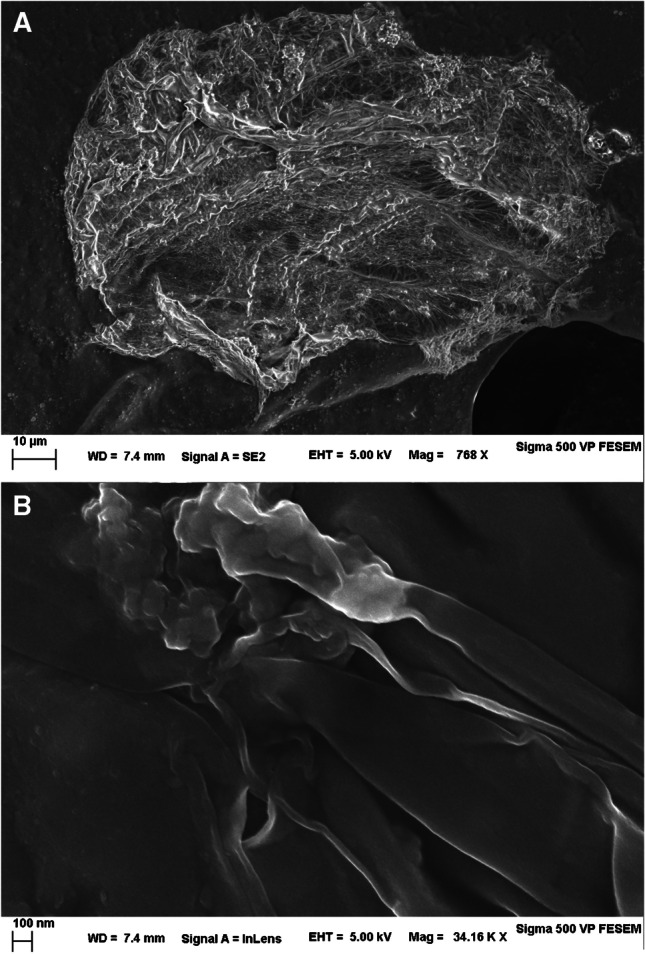
Scanning electron microscopy images of a pyrrole capsule casted onto adhesive carbon tape. The material was prepared with 25 mM FeCl_3_, 2:1 vol/vol. 10 µL were drop-casted, allowed to dry for 5 min on the bench, and vacuumed in a desiccator prior to EM. In **A**, secondary electrons (SE) image was taken at an acceleration of 5 keV, an aperture of 30 µm, and a bias of + 300 V in the SE collector. In **B**, a wrinkle on the capsule envelope was imaged at a high magnification using the InLens SE detector at a reduced aperture of 20 µm

Microfluidic chips with different channel intersection widths (10 and 30 µm) were investigated for their ability to generate polypyrrole capsules. Two programmable syringe pumps (NE-1000, New Era Pump Systems Inc.) were used to feed the pyrrole and FeCl_3_ solutions separately. Typical flow rates used were 0.4 µL min^−1^ for pyrrole and 0.10 µL min^−1^ for the FeCl_3_ solution. A 0.01 M FeCl_3_ solution was used, as it was shown to yield optimal results with the vortex-based method. Pyrrole and FeCl_3_ mixed at the intersection (Fig. 2), generating the polypyrrole capsules. To facilitate the collection and transfer of polypyrrole capsules from the collection pool, 1 µL min^−1^ DI water was run after operating the chip. The polypyrrole capsules were collected in an Eppendorf tube for particle size analysis and electrode modification.

### Preparation of polypyrrole-modified electrode

The polypyrrole capsules generated from the microfluidic devices were casted on a 4-mm carbon screen-printed electrode (SPE), DRP-C110 from Metrohm Dropsens (Llanera, Spain). 0.2 mg polypyrrole was loaded on the carbon electrode. Two microliters of 0.5% Nafion was drop-casted on the electrode and air-dried prior to electrochemical measurement.

### Preparation of GDH/poly-TBO/polypyrrole-modified electrode

A glucose biosensor was prepared by loading 0.2 mg polypyrrole on the carbon-SPE. Poly-toluidine blue (Poly-TBO) was electropolymerized by means of cyclic voltammetry. For this, the electrode was submerged in a solution containing 0.5 mM toluidine blue and 0.1 M NaNO_3_ in phosphate buffer pH 6.8. The potential of the electrode was swept from − 0.2 to 1.1 V (vs. Ag/AgCl 3 M KCl) at a scan rate of 50 mV s^−1^ for 30 cycles. The electrode was rinsed with buffer solution and air dried. Four microliters of glucose dehydrogenase from *Pseudomonas sp.* (GDH, 1 mg mL^−1^, Sigma-Aldrich) was added to the electrode surface. Once the electrode was dry, 4 µL of 0.5% Nafion was drop-casted on the top layer of the electrode. The GDH/poly-TBO/Ppy/SPE prepared was kept at 4 °C overnight before use.

### Electrochemical characterization of the modified electrodes

The electrode active surface area of the polypyrrole-modified electrode was evaluated by cyclic voltammetry from 0.6 to − 0.2 V (vs. Ag/AgCl 3 M KCl) in a solution of 5 mM K_3_[Fe(CN)_6_] and 0.1 M KCl. The electroactive surface area of the electrode was estimated from the oxidation peak in its voltammogram using the Randles–Sevcik Eq. [23]:

$${I}_{p}=2.69\times {10}^{5} {AD}^\frac{1}{2} {n}^\frac{3}{2} {v}^\frac{1}{2} C$$

where *I*_p_ is the peak current, *A* is the area of the electrode surface (cm^2^), *n* is the number of electrons transferred in the redox reaction, *D* is the diffusion coefficient (cm^2^ s^−1^), *v* is the scan rate of the electrode (V s^−1^), and *C* is the concentration of the redox species (mol L^−1^). Considering the known value for the diffusion coefficient of [Fe(CN)_6_]^3−^, 6.30 × 10^−6^ cm^2^ s^−1^, the electroactive surface area of the electrode can be estimated.

Electrochemical impedance spectroscopy (EIS) of the polypyrrole/SPE and bare carbon-SPE was performed in a redox couple solution containing [Fe(CN)_6_]^3−/4−^ in 0.1 M KCl at an open-circuit potential of 0.215 V. This allows estimating the charge transfer resistance of the electrodes, which was assessed at frequencies in the range 100,000–0.05 Hz with an AC amplitude of 5 mV.

The biocatalytic glucose oxidation activity of the NAD-GDH/poly-TBO/polypyrrole/SPE device was evaluated by chronoamperometry at a constant applied potential of 0.2 V (vs. Ag/AgCl 3 M KCl) in an electrochemical cell containing 8.0 mL air-equilibrated electrolyte (glucose in phosphate buffer pH 7.4), stirred at 350 rpm.

## Results and discussion

### Optimization of the vortex-based encapsulation

A range of synthesis variables were explored to prepare Ppy capsules using a vortex, including the effect of a surfactant, the concentration of oxidant, and the organic to aqueous phase ratio. Based on previous reports, [24–26] we expected that a surfactant might be necessary to stabilize the aqueous droplets in pyrrole while polymerization of the droplets took place. Figure S1 shows images of the dispersions prepared at increasing CTAB concentration. The results indicate that a surfactant is not necessary to create stable microcapsules. On the contrary, its presence may lead to the formation of unreacted aqueous droplets dispersed throughout the organic phase. This might be due to the difficulty of the oxidant and the pyrrole to react across the layer of surfactant.

Besides CTAB, we also evaluated the effect of other dispersants (Figure S2). Tween 20, a polysorbate-type nonionic surfactant, had a qualitatively similar effect to CTAB, leading to dark Ppy clumps and coalescence of phases. PEG6000 had the interesting effect of broadening the particle size distribution of the capsules obtained (Figure S2b). PEG is commonly used to stabilize dispersions and has been proposed to bind Ppy. [27, 28] Lastly, glycerol was found to have little effect on the droplet size distribution (Figure S2a) except perhaps an increased sphericity, which may be a result of changes in surface tension. Based on these observations, subsequent experiments were conducted in the absence of surfactant.

The concentration of oxidant (FeCl_3_) in the aqueous phase was found to be critical to produce particles with the desired characteristics. In the absence of FeCl_3_, small, stable droplets did not form. Instead, the phases separated after mixing, and slow polymerization of pyrrole occurred on the surface exposed to air (Fig. 1a). For FeCl_3_ concentrations $\le$ 10 mM, fragments of polymer film were observed, but these did not form closed capsules. On the other hand, when FeCl_3_ was introduced in the aqueous phase at concentrations $\ge$ 25 mM, stable capsules formed, with diameter around 100 µm regardless of the concentration used. At concentrations $\ge$ 100 mM FeCl_3_, dark polymeric clumps started to be observed in a concentration-dependent manner, which partitioned preferably into the aqueous phase. As FeCl_3_ concentration was further increased, the clumps started to be visible in the continuous pyrrole phase as well, resembling “pyrrole black”. [29, 30] The results in Fig. 1 indicate that 25 mM is a reasonable concentration to form stable capsules and minimize other polypyrrole aggregates.

To differentiate the aqueous and organic phases under the optical microscope, it is possible to introduce dye molecules. Trypan blue and a red food colorant were chosen, as they are highly soluble in the aqueous phase (although they may precipitate at high concentrations) and are insoluble in pyrrole. Thus, when the dyes were preloaded in the aqueous phase prior to encapsulation, coloration and formation of crystals are only seen in the disperse phase (Figure S3). This confirms that the disperse phase in the preparations is the aqueous phase.

We investigated the wall thickness of the Ppy droplets obtained. SEM images of the capsules were taken (Fig. 2). The wrinkles on the surface of the capsules suggest that the thickness of the polypyrrole wall under the chosen 25 mM FeCl_3_ conditions is likely around 5–10 nm for the vortex-based preparation method. Using a higher concentration of FeCl_3_ (100 mM) did not seem to substantially impact the thickness (Figure S4), suggesting that the Ppy layer limits the mixing and polymerization reaction as it grows. Interestingly, at higher FeCl_3_ concentration, we observed nanoparticles dispersed throughout the Ppy layer (Figure S4b). Elemental analysis using EDX was conducted on selected areas of the droplets (Figure S5). A beam acceleration of 12 keV was used aiming to increase the spatial resolution to the sub-micron range while maintaining sufficient excitation of iron (Fe K_α_ = 6.4 keV). The results indicated a variable Fe/Cl ratio depending on the area, suggesting that these nanoparticles might be some type of iron oxide or salt.

On the other hand, we studied a range of aqueous to organic phase volume ratios while keeping constant the total preparation volume. The results, shown in Figure S6, evidence that a broad range of ratios can be used. Capsules could be reliably produced with organic: aqueous ratios as low as 1.0 (for 25 mM FeCl_3_) or higher than 100, which offers flexibility in the preparation depending on the intended use. For example, low values may be appropriate to maximize the interfacial area while reducing the use of monomer, whereas having some unreacted monomer left may facilitate subsequent casting and polymerization of the particles on a support. Once prepared, the capsules remained stable over time, both in the preparation mixture and after casting, which is a desirable characteristic to stably modify electrodes. Thus, we next focused on reducing the size of the droplets to maximize their specific surface area. For this, we explored the application of this chemistry in a microfluidic device.

### Microfluidic generation of polypyrrole microcapsules

**Fig. 3:**
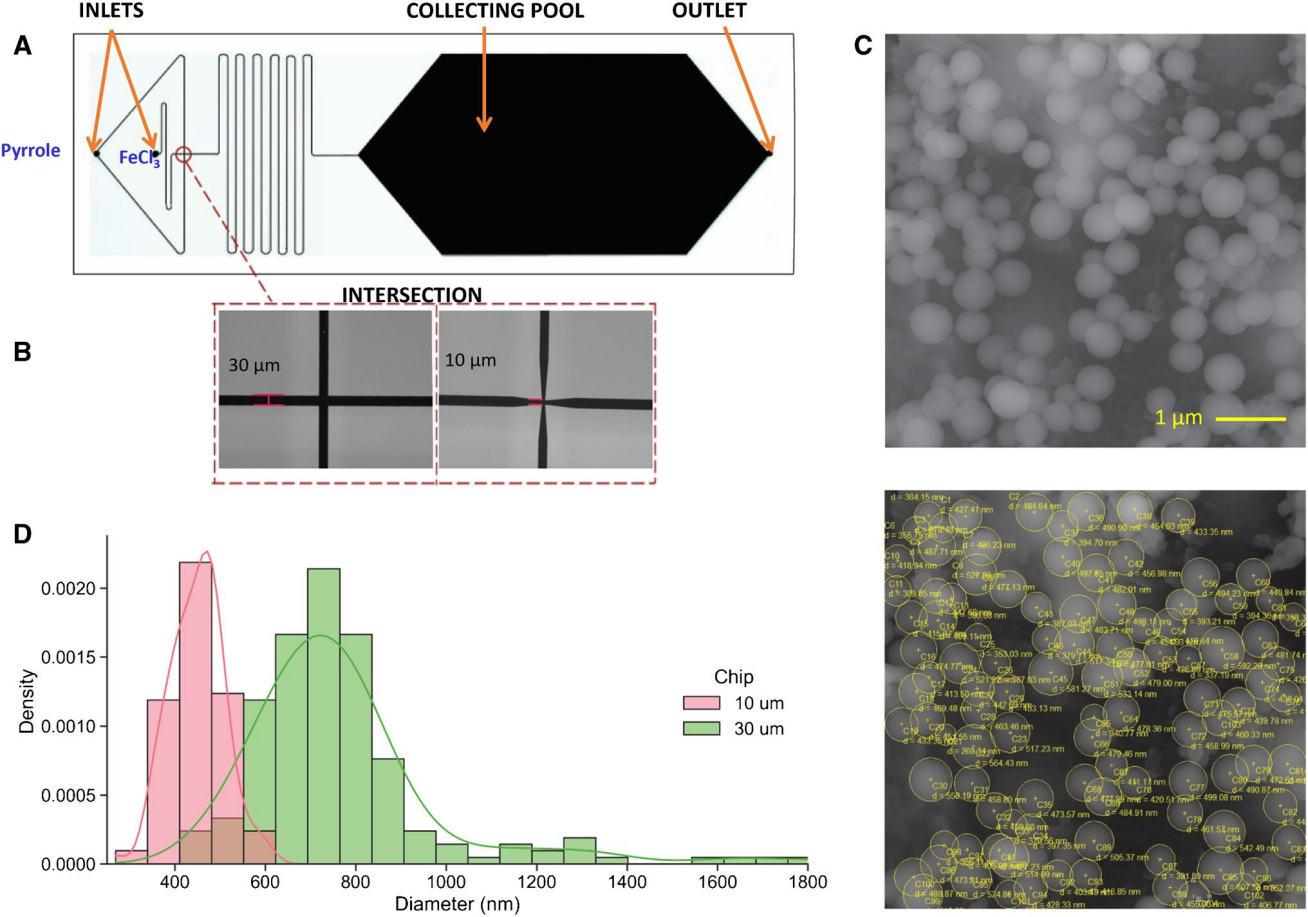
The compatibility of the polypyrrole encapsulation by chemical oxidation with microfluidic chips was demonstrated. **A** Layout of the microfluidic chips fabricated, spanning inlets for the pyrrole and aqueous phases, a cross-shaped intersection where the two solutions are mixed, a zig-zag polymerization region, a collecting pool, and an outlet. **B** Two different intersection sizes of 30 and 10 µm were fabricated. **C** The generation of polypyrrole microcapsules using the microfluidic chips was demonstrated. **D** The chips produced a narrow particle size distribution (PSD), and the size of the particles could be modulated by the width of the channel intersection. The PSD was evaluated by analyzing multiple images of the product of each chip using ImageJ (*n* > 100)

Microfluidics is becoming an increasingly affordable and scalable technology to carry out flow chemistry and prepare materials with superior control over the spatial dimensions. A variety of droplet-generation microfluidic chips have been proposed. [31–33] We explored the compatibility of the chemical encapsulation procedure reported above with a custom-made microfluidic chip design. Two variations of the device were manufactured seeking to affect the size of the droplets generated. These are illustrated in Fig. 3. To simplify the fabrication process, all chips were prepared with a constant channel height of 50 µM.

The results of using the chip are shown in Fig. 3c and d. These results demonstrate the ability to produce Ppy microcapsules using the microfluidic chip with a fine control of the particle dimensions and a narrow particle size distribution.

### Casting the microcapsules on an electrode

**Fig. 4:**
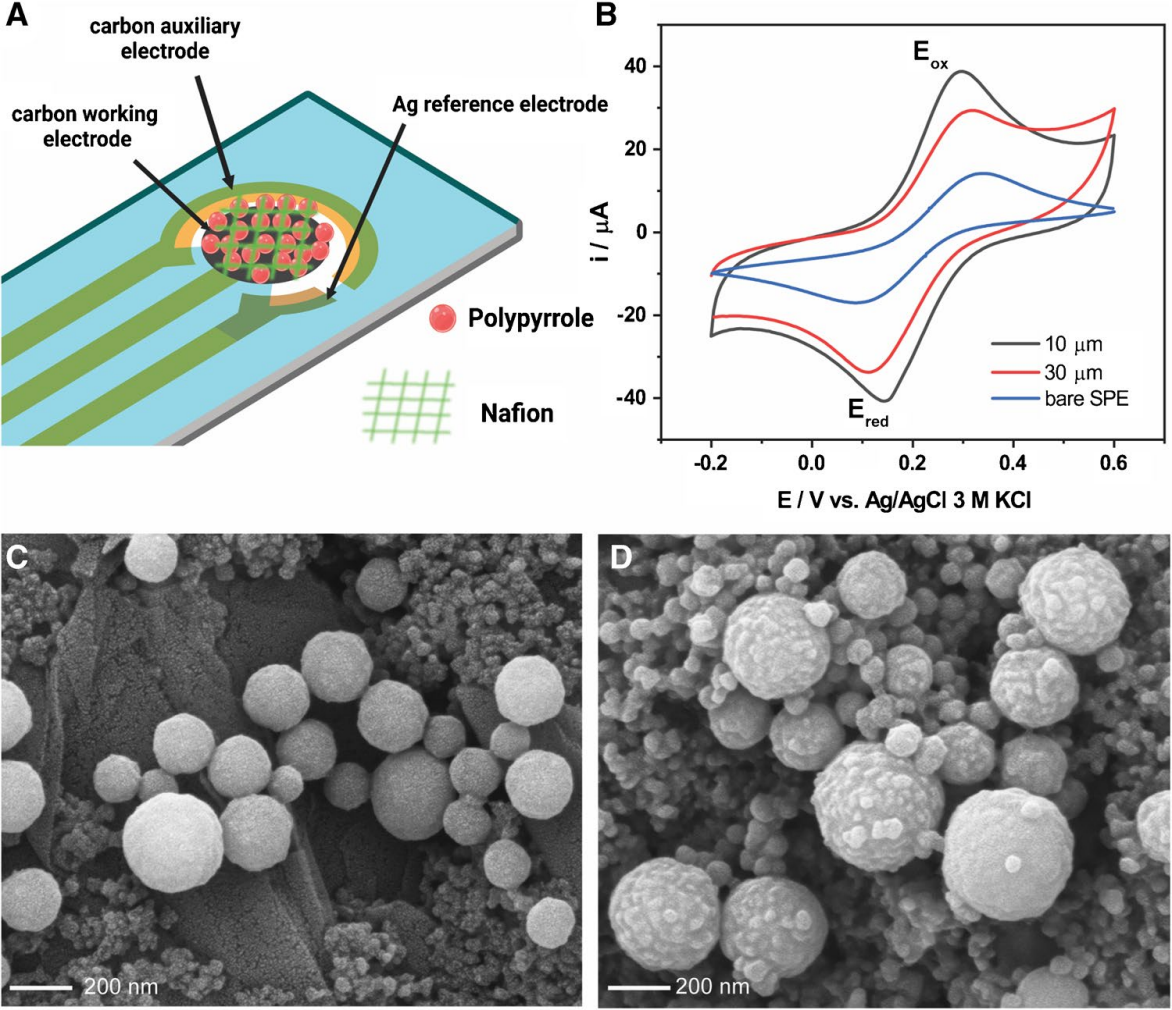
Polypyrrole microcapsules are used to modify a carbon screen-printed electrode (SPE) and increase its electroactive surface area. **A** A carbon SPE was modified by co-depositing the polypyrrole microcapsules previously prepared (Fig, 3). **B** Cyclic voltammograms of a bare carbon SPE and polypyrrole modified SPEs in 5 mM K_3_[Fe(CN)_6_] in 0.1 M KCl at the scan rate of 100 mV s^−1^. The modification provides a dramatic increase in electroactive surface area and Nafion. SEM images were acquired for the electrodes modified with microcapsules prepared with the 10 µm **C** and the 30 µm **D** microfluidic chip at 50,000 magnification (2 nm gold sputtering was applied in this case to improve the imaging resolution). The capsules remain intact during the immobilization. Panel **A** created with BioRender.com

Polypyrrole is widely used in electrochemical devices, including biosensors, supercapacitors, and electrocatalysts. [2] This is due to its favorable properties, highlighted in the introduction, such as electrical conductivity and biocompatibility. To investigate the utilization of Ppy microcapsules in an electrochemical device, the microcapsules were first drop-casted on carbon SPE electrodes, along with a small amount of Nafion to improve their fixation (see Methods). The modified electrodes were evaluated for their electroactive surface area in a solution of K_3_[Fe(CN)_6_]. The cyclic voltammograms of the SPEs with different modifications are shown in Fig. 4. The electrodes with Ppy microcapsules showed a significant increase in the peak oxidation current, and therefore electroactive surface area, compared to the unmodified carbon SPE.

On the other hand, the peak-to-peak separation in the voltammogram can inform about the redox reversibility of the redox couple [Fe(CN)_6_]^3−/4−^. The large peak-to-peak separation observed in the unmodified SPE indicates a sluggish electron transfer. By contrast, a much smaller peak-to-peak separation was observed for the electrode modified with the smallest Ppy capsules. The peak current, peak-to-peak separation, and electroactive surface area of the electrode with different modifications are shown in Table S2.

**Fig. 5:**
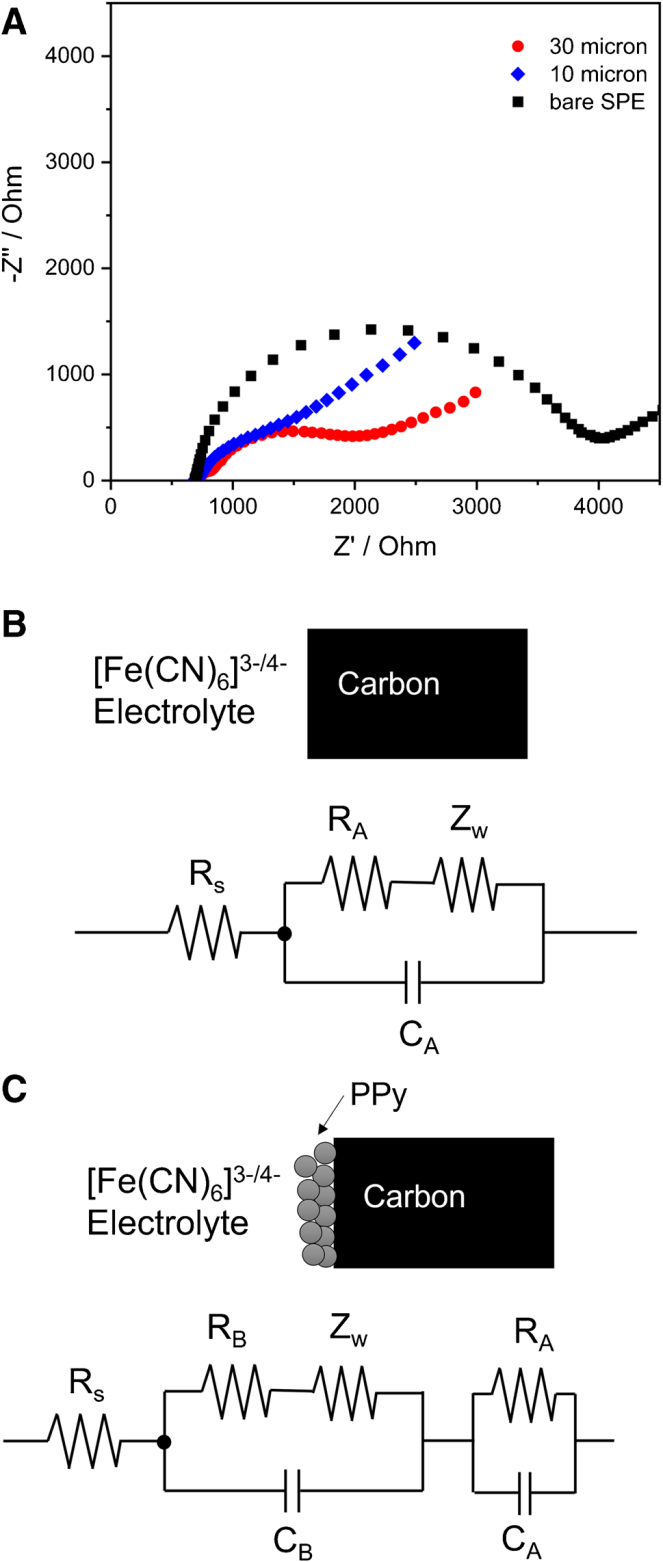
**A** Nyquist diagram of the electrodes with different modifications in the presence of the redox couple 5 mM [Fe(CN)_6_]^3−/4−^ in 0.1 M KCl. DC potential: 0.215 V vs. Ag/AgCl 3 M KCl, AC amplitude of 5 mV with a frequency range from 100 kHz to 50 mHz. **B** Equivalent circuit for the bare SPE electrode. **C** Equivalent circuit for the Ppy/SPE electrode

The Ppy-modified SPEs were investigated for their charge transfer resistance (*R*_ct_) by EIS in the electrolyte containing the redox couple [Fe(CN)_6_]^3−/4−^. Nyquist plots of the Ppy/SPEs and the unmodified SPE are shown in Fig. 5. The results reveal the impact of polypyrrole in increasing the electrical conductivity. This is indicated by the semicircle in the Nyquist plot becoming smaller compared to that for the bare SPE, which shows the largest electron transfer resistance. When Ppy particles with the smaller size distribution (10 µm chip) were immobilized on the SPE, the *R*_ct_ decreased, and the conductivity increased compared to the immobilization of larger particles. This points to an improved accessibility of the redox couple to the electroactive surface and a shorter distance for electron conduction to the electrode surface.

Quantitative parameters can be derived from EIS by modeling the electrochemical system (Table S2). Appropriate equivalent circuits need to be chosen for the electrode with different modifications. [34] For a bare SPE, the Randles circuit was selected as shown in Fig. 5B. By contrast, for the Ppy/SPE, additional elements had to be introduced in the equivalent circuit, as shown in Fig. 5C. The results indicate increased double layer capacitance values for both Ppy-modified electrodes, reflecting the capacitive properties of Ppy.

### Polypyrrole-coated electrodes as glucose biosensors

**Fig. 6:**
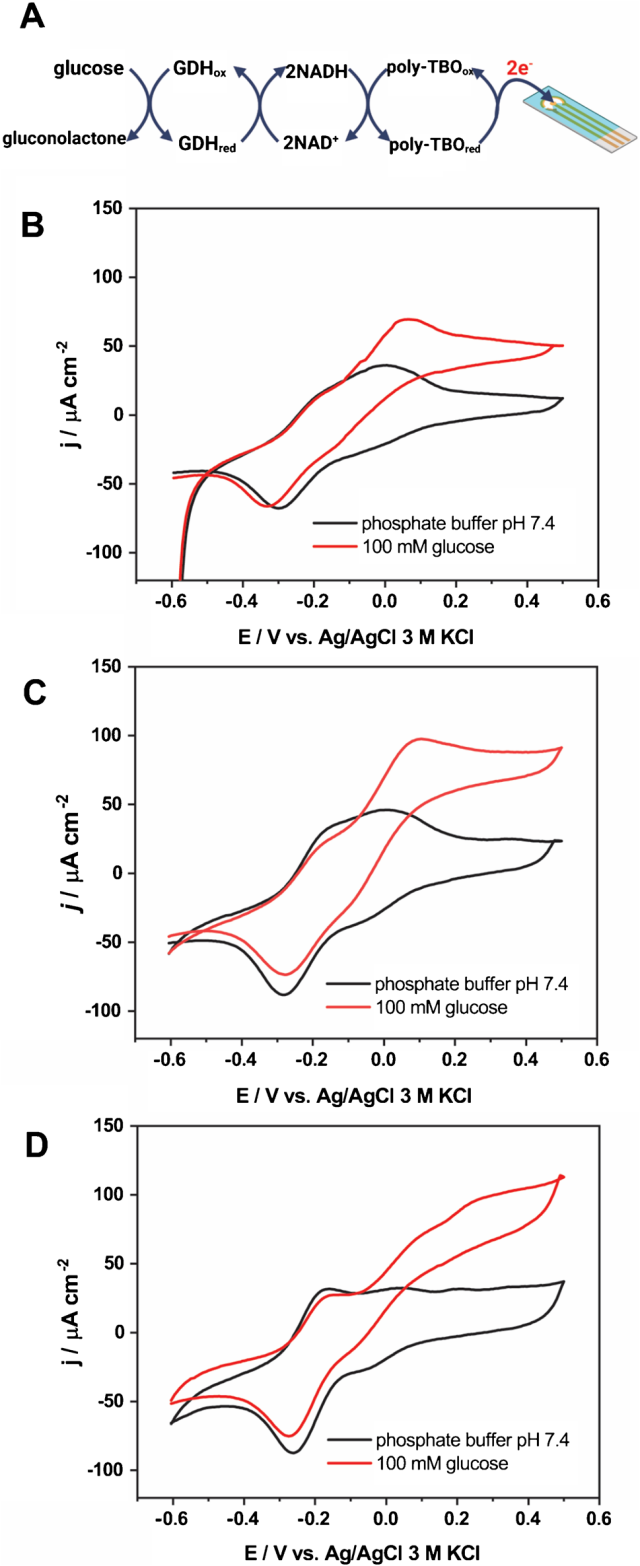
A glucose biosensing electrode was prepared by modifying the polypyrrole/SPE (Fig. 4) with glucose dehydrogenase (GDH) and poly-TBO for NAD^+^ regeneration. **A** Electron transfer pathway of the GDH/poly-TBO/SPE glucose biosensor designed based on mediated electron transfer (MET) mechanism. Cyclic voltammograms of GDH/poly-TBO/SPE **B** and GDH/poly-TBO/polypyrrole/SPE (polypyrrole capsules from the 10 µm chip in (**C)** and 30 µm chip in **D** in the presence of 0.1 M phosphate buffer pH 7.4 containing 3 mM NAD^+^ in 100 mM glucose (red) and the absence of glucose (black); scan rate = 5 mV/s, air-equilibrated electrolyte. Panel **A** created with BioRender.com

Next, we explored the fabrication of a glucose biosensor by leveraging the modified electrodes developed above. Polypyrrole is an excellent material for the immobilization of redox enzymes in electrochemical biosensors. [35, 36] The enzyme glucose dehydrogenase (GDH) catalyzes the oxidation of glucose in the presence of the cofactor NAD^+^. Thus, to ensure the continuous operation of the biosensor, poly-TBO was co-immobilized with GDH to regenerate NADH into NAD^+^ (Fig. 6A). [37, 38] Three electrodes were prepared: GDH/poly-TBO/SPE, GDH/poly-TBO/Ppy (30 µm chip)/SPE, and GDH/poly-TBO/Ppy (10 µm chip)/SPE. Note that the latter two electrodes were prepared by co-immobilizing poly-TBO and GDH on the Ppy/SPE electrodes described in the previous section. Also, the same load of GDH and poly-TBO was used in all cases (see Methods).

The performance of the three electrodes was evaluated by cyclic voltammetry in the presence and absence of glucose in the medium. The results, shown in Fig. 6B–D, evidence an increased current in the presence of glucose due to the redox catalysis. The electrode modified with the Ppy from the 10 µm chip shows a higher catalytic current in the presence of glucose than the unmodified electrode. As studied above, the introduction of Ppy microcapsules provides an increased surface area, which may improve enzyme dispersion and electron transfer.

Interestingly, the oxidation peak for the electrode modified with Ppy from the 10 µm chip shows a relatively lower potential than that with Ppy from the 30 µm chip. This indicates that regeneration of NAD to NADH takes place at lower potential in the former. This might be associated with the smaller size of Ppy allowing a faster electron transfer. It may also be due to subtle differences in the wall thickness of the capsules in both preparations.

**Fig. 7:**
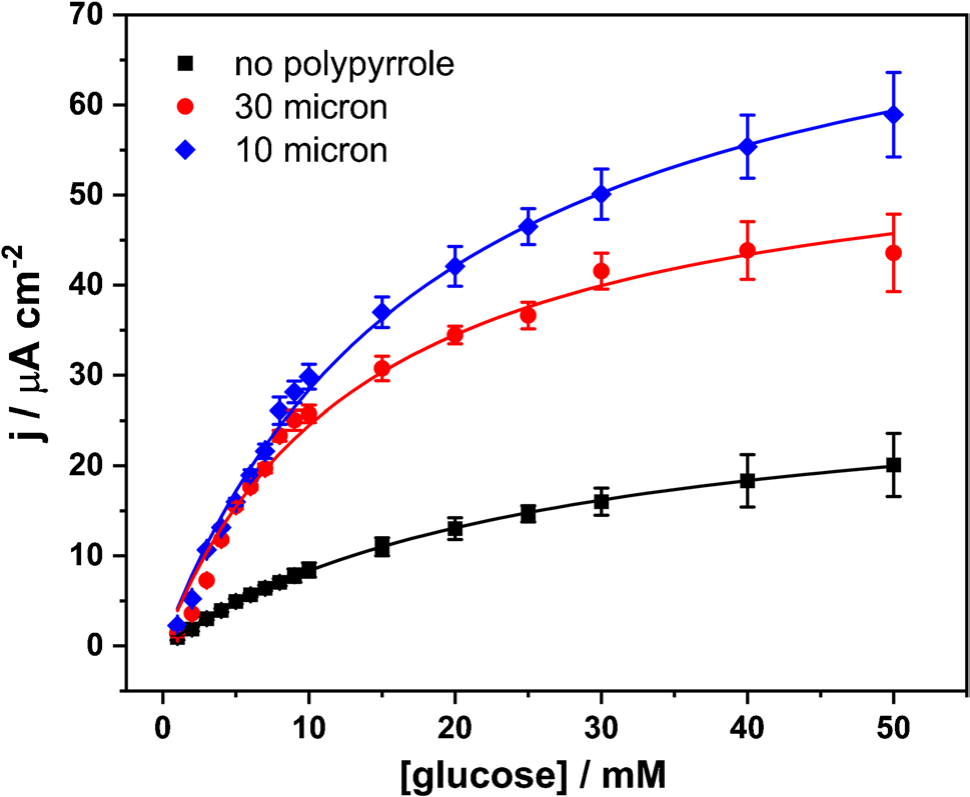
The proposed biosensors with polypyrrole microcapsules showed a superior dynamic range for the determination of glucose. Chronoamperometry was carried out at a constant applied potential of 0.2 V (vs. Ag/AgCl 3 M KCl) in an electrochemical cell containing 8 mL air-equilibrated phosphate buffer pH 7.4, stirred at 350 rpm. A GDH/poly-TBO/SPE electrode was used as the “no-polypyrrole” control. Results were fitted to Michaelis–Menten kinetic expressions by nonlinear regression, which can be used as calibration curves for glucose analysis

Chronoamperometric measurements of the three electrodes were carried out at varying glucose concentrations, as a potential biosensing strategy. The results in Fig. 7 show that the incorporation of the polypyrrole capsules leads to a broader dynamic range and a higher sensitivity for the sensors. The response curves for all three electrodes are well described using Michaelis–Menten kinetics and can be used as calibration curves. The *K*_m_ values are 26.7, 18.7, and 13.9 mM for GDH/poly-TBO/SPE, GDH/poly-TBO/Ppy (30 µm chip)/SPE, and GDH/poly-TBO/Ppy (10 µm chip)/SPE, respectively. The lowest apparent *K*_m_ values are obtained for the smallest Ppy particles, which points to a more efficient electron harvesting from the glucose substrate in this design. The GDH/poly-TBO/Ppy (10 µm chip)/SPE offers a linear analytical range of 1.0–9.0 mM (*R*^2^ = 0.993). The limit of detection obtained was 0.09 mM (estimated from 3 s.d. of blank/slope). For reference, the normal glucose range in human urine is 0 to 0.8 mM, [39] with higher values pointing to conditions such as diabetes, pregnancy, or renal glycosuria. The reproducibility of this sensor design was evaluated by measuring five independently prepared electrodes in the presence of 5 mM glucose and resulted in an excellent relative standard deviation (RSD) of 3.6%.

The advantages of the glucose sensor proposed include a simple fabrication, suitable analytical range, and good reproducibility. Moreover, basing the sensor on GDH enzyme ensures a good selectivity toward glucose oxidation. [40] One limitation of the sensor, however, is the limited storage stability of wild-type GDH. Table 1 surveys some electrochemical glucose sensors proposed in the recent literature.

**Table 1:** Overview of recent nanomaterial-based electrochemical sensors for glucose determination. *DPV*, differential pulse voltammetry; *ECL*, electrochemiluminescence; *CV*, cyclic voltammetry; *Amp*, amperometry; *TR*, thermal resistance

Materials	Detection approach	Linear range (M)	LOD (M)	Ref
MIP/TNO@NC/GCE	DPV	1.0 × 10^−5^ to 5.0 × 10^−3^	1.0 × 10^−5^	[41]
Ppy MIP/Electrospun Nylon Fibers	TR	1.0 × 10^−4^ to 1.0 × 10^−3^	1.0 × 10^−4^	[42]
Ppy/Plu/C_3_N_4_-Ni(OH)_2_/GOx	ECL	0.5 × 10^−6^ to5.0 × 10^−4^	4.0 ×10^−8^	[43]
Ppy/AuNPs_(AuCl4-)_-GOx/GR	CV	1.0 × 10^−4^ to 7.0 × 10^−4^	1.0 × 10^−4^	[44]
AgNPs@GQDs/CS/GCE	CV	1.0 × 10^−4^ to 1.0 × 10^−2^	1.0 × 10^−5^	[45]
NiO@Ppy/Au/GCE	CV	0.5 × 10^−6^ to 1.7 × 10^−3^	1.5 × 10^−7^	[46]
AuNPs/(Ni–Fe)-PBA/Nafion/Au-wire	Amp	1.0 × 10^−5^ to 1.6 × 10^−2^	4.7 × 10^−6^	[47]
NAD-GDH/poly-TBO/Ppy/SPE	Amp	1.0 × 10^−3^ to 9.0 × 10^−3^	9.0 × 10^−5^	This work

The proposed glucose sensor was tested for its applicability for glucose analysis in synthetic urine (Sigma-Aldrich Sigmatrix), a non-biological electrolyte that simulates human urine. Glucose was spiked in to attain a concentration of 1.0 mM. A satisfactory recovery percentage was obtained with the proposed sensor, as shown in Table S3. The synthetic urine containing 1.00 mM glucose was also measured with a commercial glucometer (Roche Accu-Chek), and the value obtained was 1.0 ± 0.1 mM, which is approximately similar to the value from the proposed sensor.

## Conclusions

Polypyrrole is one of the most widely used polymers in electrochemical research. To spread its use in commercial devices, it is crucial to reduce the cost and improve the sustainability of the manufacturing process. This includes reducing the use of energy, co-solvents, and expensive templating molecules. In this work, we report a method for the preparation of polypyrrole capsules of variable sizes. The method proposed is carried out at room temperature, with short synthesis times, and without co-solvents or templating molecules.

The method was first demonstrated with a benchtop vortex. Exploring different variables provided a suitable range of monomer to oxidant and phase ratios. The capsules generated by this route had sizes in the 10^2^ µm range and wall thicknesses in the low-nanometer range. Next, the synthesis was adapted for use in a custom-made microfluidic chip. The adaptation was successful and allowed almost 3 orders of magnitude reduction in particle size, down to the 10^2^ nm size range. By varying the geometry of the microfluidic chip, we also show that it is possible to affect the particle size distribution. Intermediate particle sizes between these two methods might be achievable, for example, by increasing the mechanical energy input in the benchtop method or by further tuning the geometry of the microfluidic chip. The approach might be compatible with other oxidants, dopants, concentrations, or flow rates, which might also affect the particle size distribution, the thickness of the capsules, or the efficiency of the process. Some shortcomings of the technology include the cost of developing a microfluidic device as well as the potential clogging of the chip after repeated, intermittent operation. The latter could be mitigated with optimized chip materials and flushing protocols.

The particles generated by the microfluidic route were used to modify an enzyme-based glucose biosensor. Other things being equal, the incorporation of polypyrrole particles led to a substantial increase in electroactive surface area and a remarkable increase in the dynamic range of the glucose sensor. The modification with the polypyrrole particles described here could likely help improve the performance of many other bioelectrode systems. The strategy shown in this work might thus help manufacture high-performance, low-cost electrochemical devices, and biosensors, and might also enable new directions in microencapsulation and molecular release.

## Supplementary information

Below is the link to the electronic supplementary material.

Supplementary file1 (PDF 1.61 MB)
