# Supplementary material for: A facile method for generating polypyrrole microcapsules and their application in electrochemical sensing
Source: Mikrochim Acta. 2022 Oct 8;189(11):410. doi: 10.1007/s00604-022-05512-1 (PMC9547802; doi:10.1007/s00604-022-05512-1)
Supplement: Supplementary file 1 — Supplementary file1 (PDF 1.61 MB) [file 604_2022_5512_MOESM1_ESM.pdf]

SUPPORTING INFORMATION

A facile method for generating polypyrrole microcapsules and their application in electrochemical sensing

Piyanut Pinyou[a,*,**], Vincent Blay[b,*,**], Jirawan Monkrathok[c,d], Pattanaphong Janphuang[c],
Kantapat Chansaenpak[e], Jaruwan Pansalee[a], Sireerat Lisnund[f]

[a]School of Chemistry, Institute of Science, Suranaree University of Technology, 111 University Ave., Nakhon Ratchasima 30000, Thailand.

[b]Department of Microbiology and Environmental Toxicology, University of California at Santa Cruz, Santa Cruz, CA, 95064, USA.

[c]BL 6: Deep X-Ray Lithography, Synchrotron Light Research Institute, 111 University Ave., Nakhon Ratchasima 30000, Thailand.

[d]Institute of Research and Development, Suranaree University of Technology, 111 University Ave.., Nakhon Ratchasima 30000, Thailand.

[e]National Nanotechnology Center, National Science and Technology Development Agency, Thailand Science Park, Pathum Thani 12120, Thailand.

[f]Department of Applied Chemistry, Faculty of Science and Liberal Arts, Rajamangala University of Technology Isan, 744, Suranarai Rd., Nakhon Ratchasima 30000, Thailand.

*First co-authors.

**Co-corresponding authors: piyanutp@sut.ac.th (P.P.), vroger@ucsc.edu (V.B.).

ORCID: 0000-0001-7978-1898 (P.P.), 0000-0001-9602-2375 (V.B.), 0000-0002-5462-9897 (K.C.).

Table S1. Conditions explored for the preparation of materials using the vortex method. In all cases the polymerization took place at room temperature.

Preparation Id.	Aqueous [$FeCl_3$] (M)	Surfactant	Aqueous [Surfactant]	Volume ratio organic:aqueous	Notes
C0	0.5	-	-	10	Figure S1
C1	0.5	CTAB	0.05 M	10	Figure S1
C2	0.5	CTAB	0.1 M	10	Figure S1
C3	0.025	Glycerol	10%v/v	2	Figure S2
C4	0.025	PEG6000	10%v/v	2	Figure S2
C5	0.025	Tween20	10%v/v	2	Figure S2
C6	0.025	-	-	10	Figure S3
O1	0.025	-	-	50	Figure S6
O2	0.025	-	-	10	Figure S6
O3	0.025	-	-	5	Figure S6
O4	0.025	-	-	3	Figure S6
O5	0.025	-	-	2	Figure S6, Figure 2
O6	0.025	-	-	1	Figure S6
O7	0.025	-	-	0.33	Figure S6
O8	0.025	-	-	0.05	Figure S6
X1	0.100	-	-	2	Figure S4, Figure S5
F0	0	-	-	10	Figure 1
F1	0.025	-		10	Figure 1
F2	0.05	-	-	10	Figure 1
F3	0.1	-	-	10	Figure 1
F4	0.25	-	-	10	Figure 1
F6	1.0	-	-	10	Figure 1

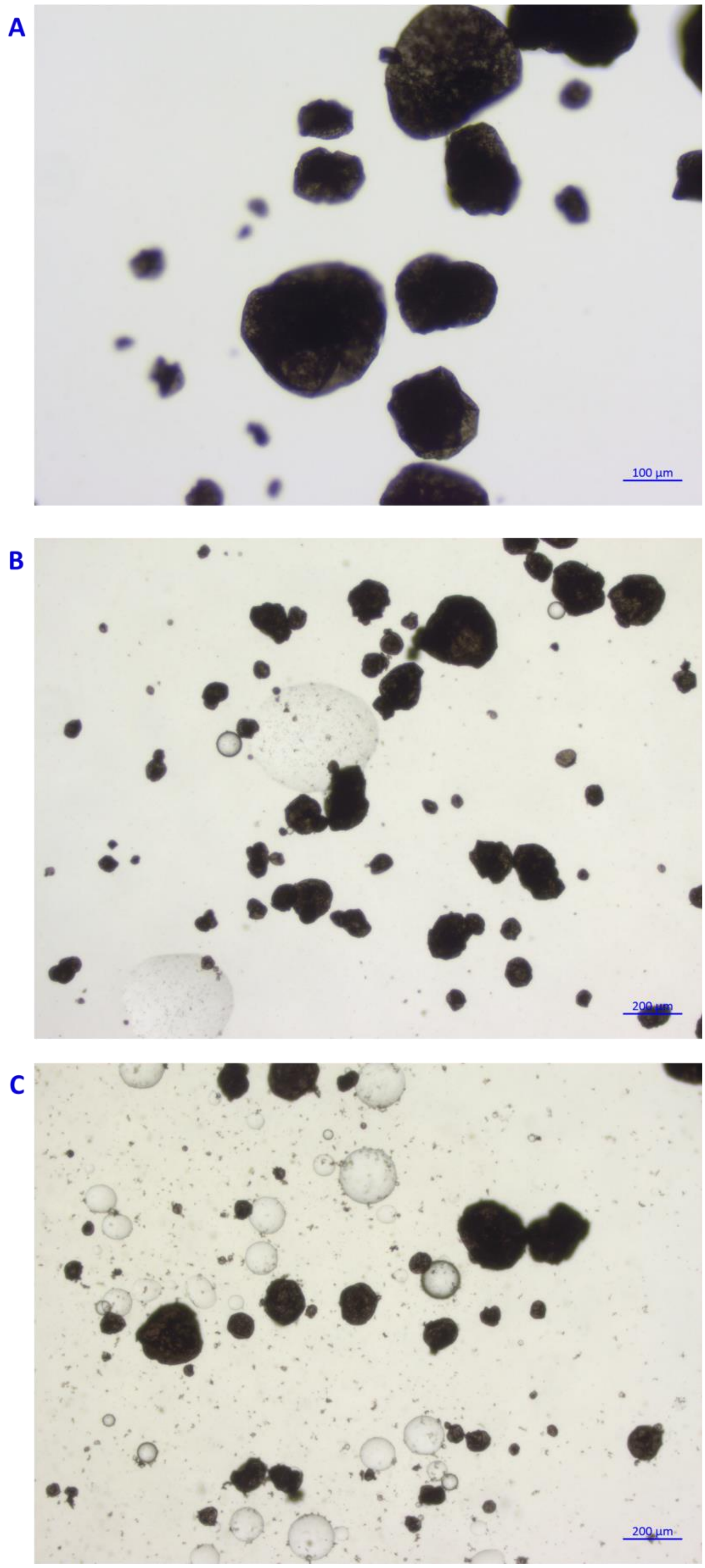

Figure S1. Effect of surfactant concentration: a) no CTAB (id:C0), b) 0.05 M CTAB (id:C1), c) 0.1 M CTAB (id:C2). In the three cases $FeCl_3$ was held constant at 0.5 M, and the organic:aqueous ratio at 10:1 vol/vol.

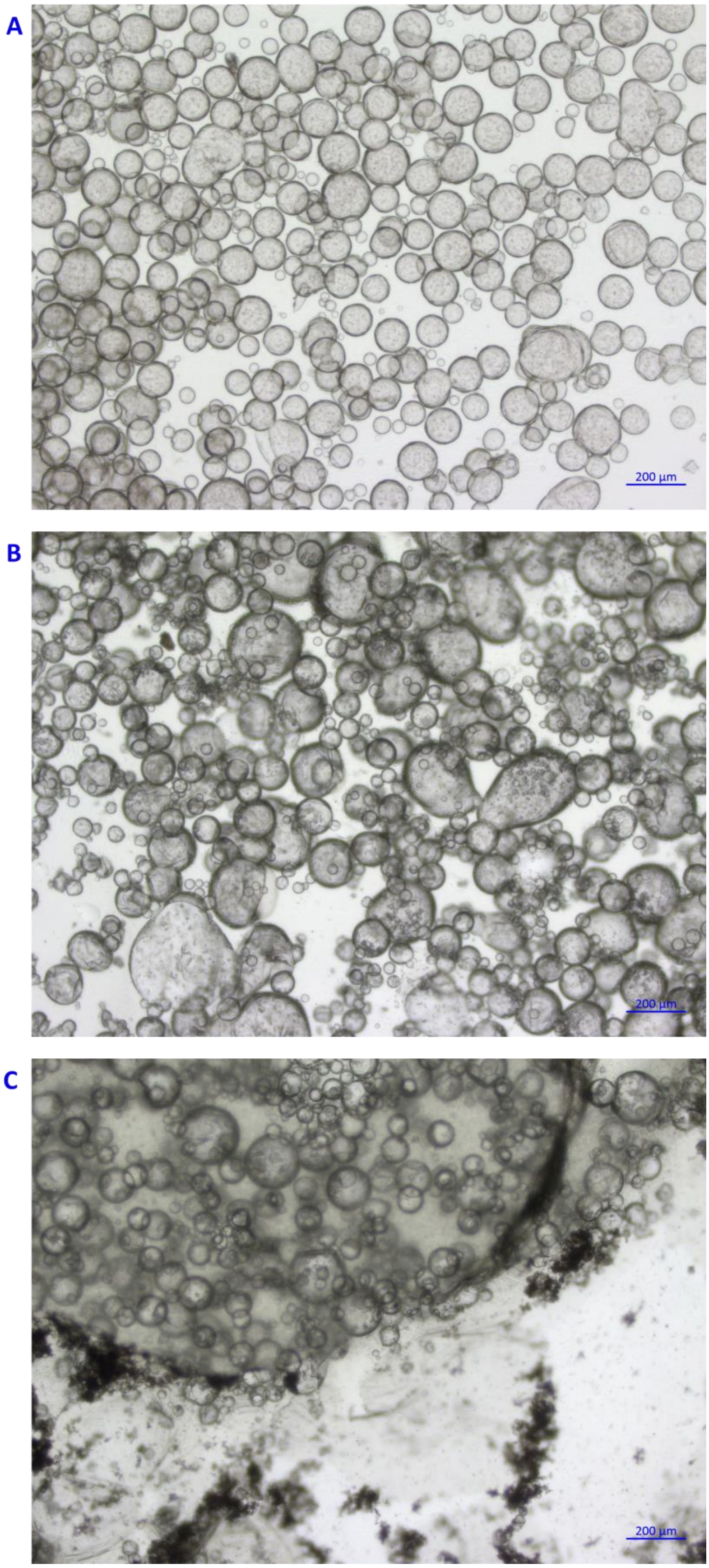

Figure S2. Effect of surfactant type a) glycerol, b) PEG6000, c) Tween20. 25 mM $FeCl_3$ concentration and organic:aqueous volume ratio 2:1 vol/vol was used in all cases. The concentration of surfactant was around 10%v/v of the aqueous phase.

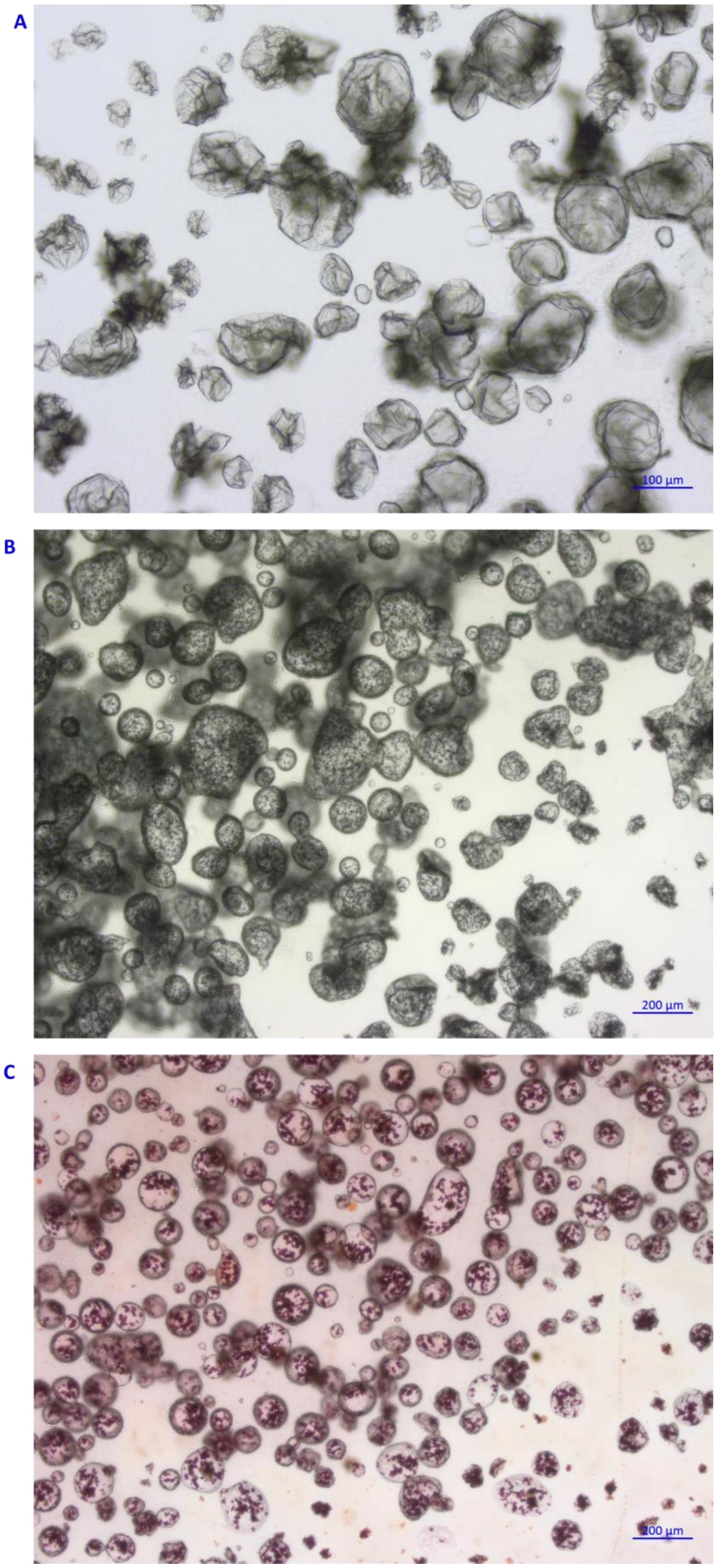

Figure S3. Demonstrating the dispersion of the aqueous phase in pyrrole: a) no dye, b) trypan blue, c) Allura Red AC and erythrosine mix. 25 mM $FeCl_3$ and organic:aqueous volume ratio 10:1 was used in all cases.

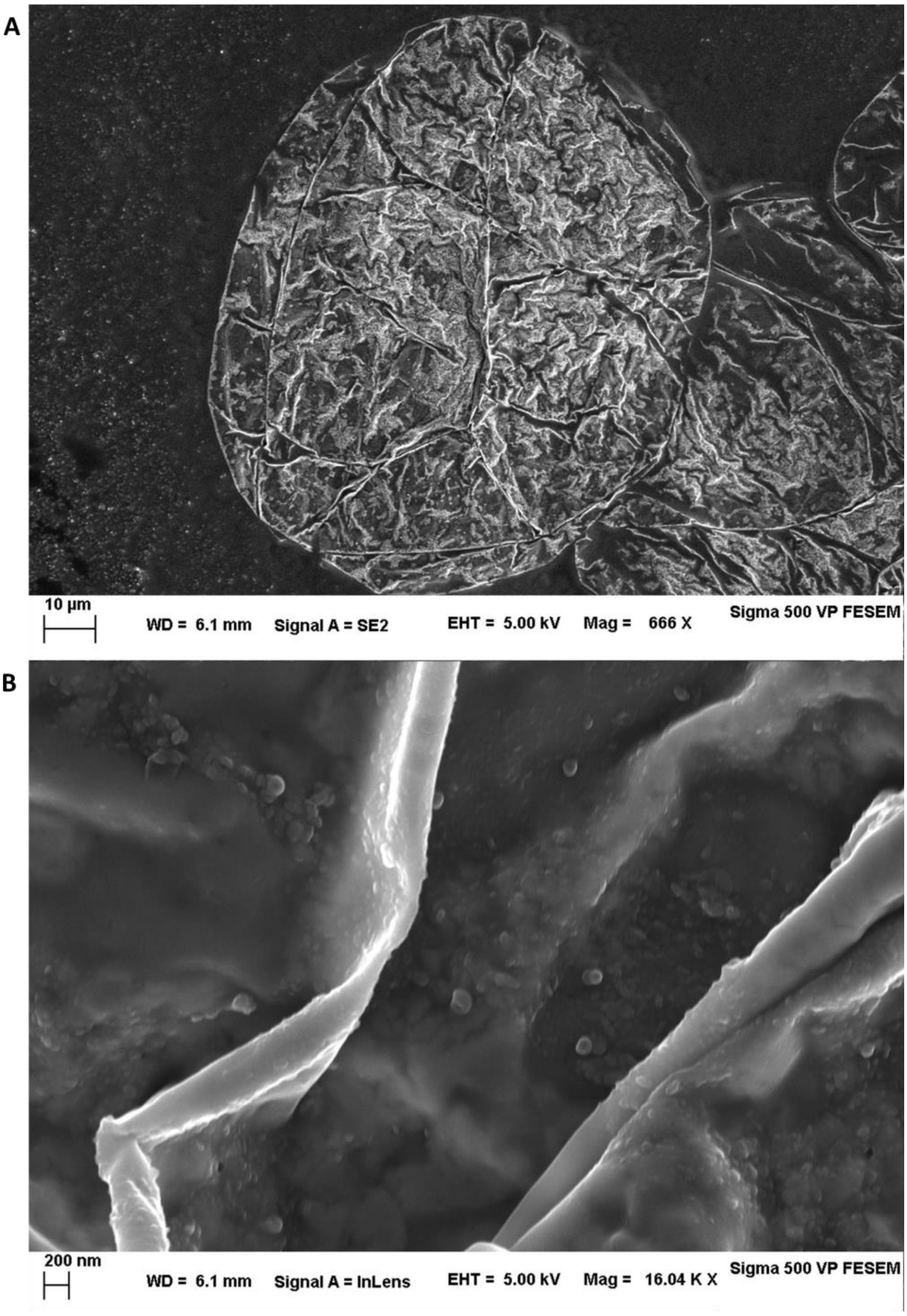

Figure S4. Scanning electron microscopy images of a pyrrole capsule casted onto adhesive carbon tape. The material was prepared according to the formulation X1 in Table S1 (100 mM $FeCl_3$, 2:1 vol/vol). 10 µl were drop-casted, allowed to dry for 5 minutes on the bench, and vacuumed in a desiccator prior to EM. In a) secondary electrons (SE) image was taken at an acceleration of 5 keV, an aperture of 30 µm, and a bias of +300 V in the SE collector. In b) a wrinkle on the capsule envelope was imaged at a high magnification using the InLens SE detector at a reduced aperture of 20 µm.

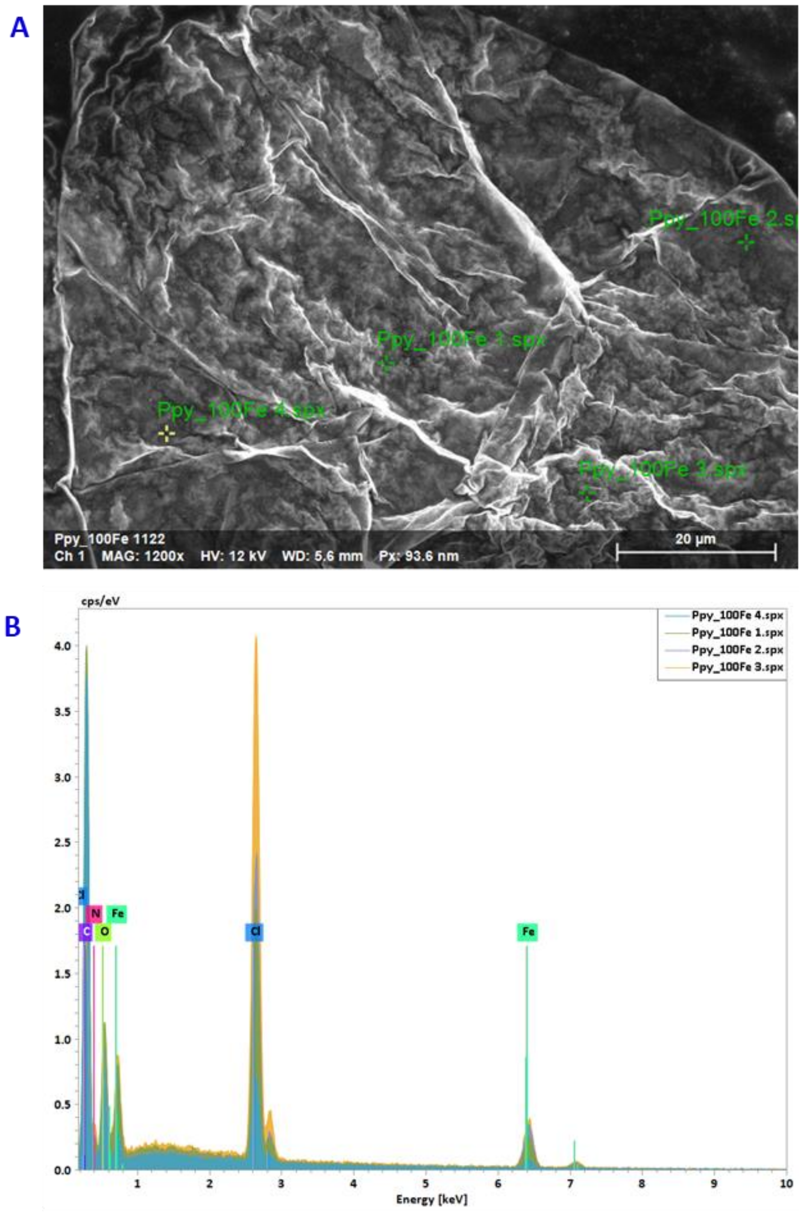

C

ROI	C (at.%)	N (at.%)	O (at.%)	Cl (at.%)	Fe (at.%)
Ppy_100Fe_1	64.6	13.5	14.3	4.4	3.2
Ppy_100Fe_2	57.9	14.1	14.2	7.7	6.2
Ppy_100Fe_3	57.7	15.9	9.09	11.4	5.9
Ppy_100Fe_4	66.2	15.1	14.9	2.3	1.5

Figure S5. EDX analysis of microcapsules. The preparation corresponds to that in Figure S5. Acceleration was limited to 10keV to improve surface sensitivity and throughput was limited to 30 kcps to improve energy resolution of light elements. a) Regions of interest analyzed. b) Representative raw EDX spectra acquired. c) Results of elemental peak deconvolution after background subtraction.

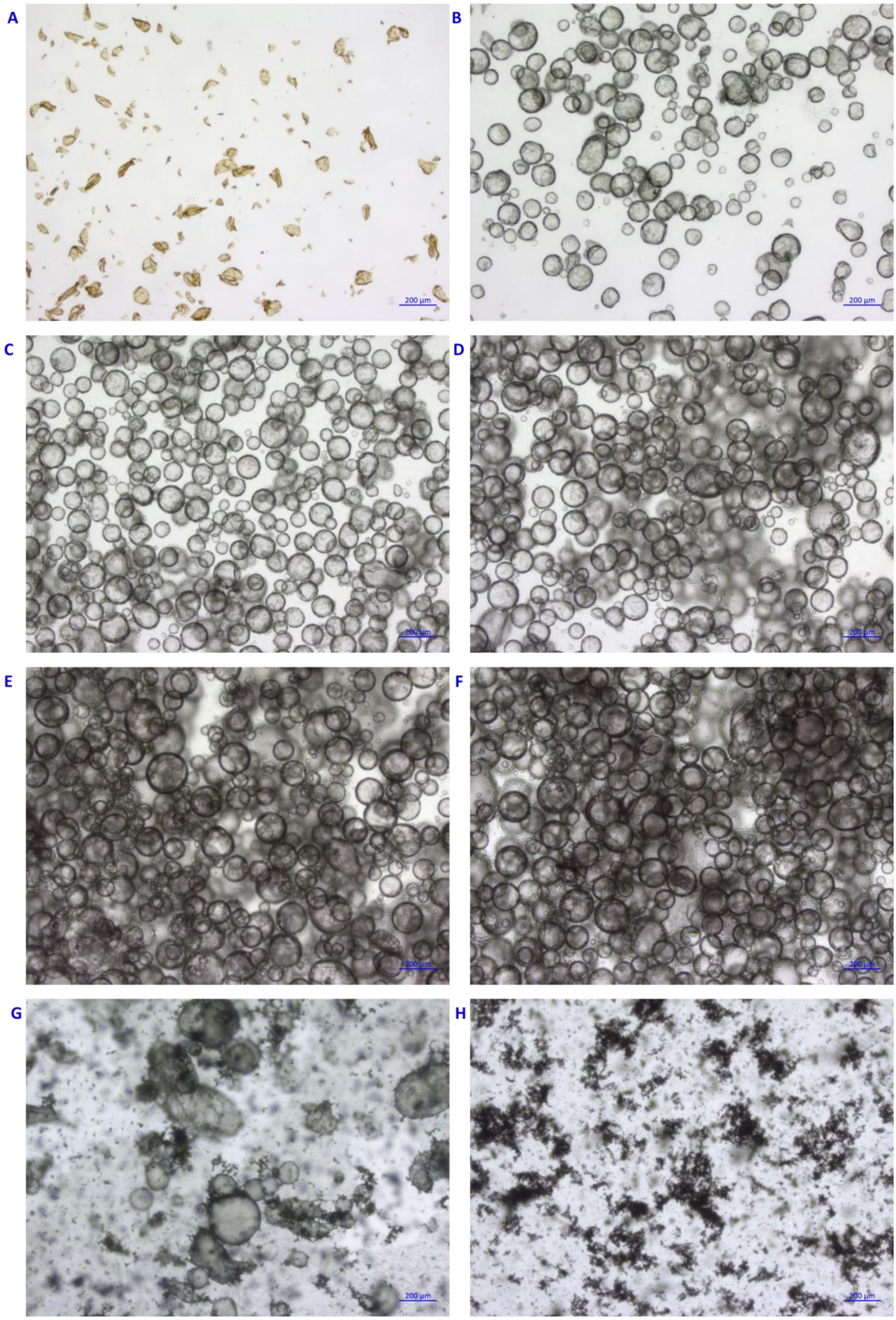

Figure S6. Effect of the organic:aqueous vol/vol ratio on the capsule preparation: a) 50:1, b) 10:1, c) 5:1, d) 3:1, e) 2:1, f) 1:1, g) 1:3, h) 1:20. The concentration of oxidant in the aqueous phase was 25 mM in all cases.

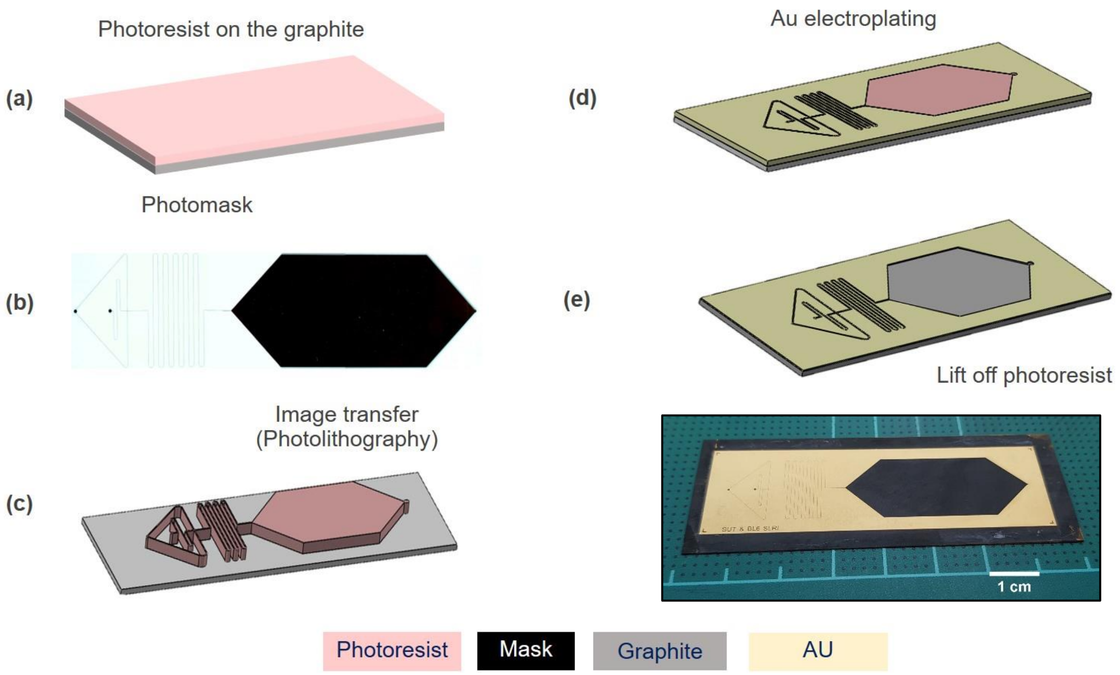

Figure S7. Schematic diagram of the microfabrication process of the X-ray gold mask. (a) The 15 μm-thick AZp4620 photoresist was spin-coated on a graphite substrate and a soft-baked was done on a hot plate at 95 °C for 5 min. (b) The photomask was placed on photoresist under a UV exposure dose of 240 mJ cm^{-2}. (c) The pattern was developed by solvent washing. (d) Forward electroplating performed at 0.5 mA cm^{-2} for 6 days creating a thin gold layer on the graphite. (e) AZp4620 photoresist was removed with a mixture of 400k AZ-developer : deionized water (1:4 by volume).

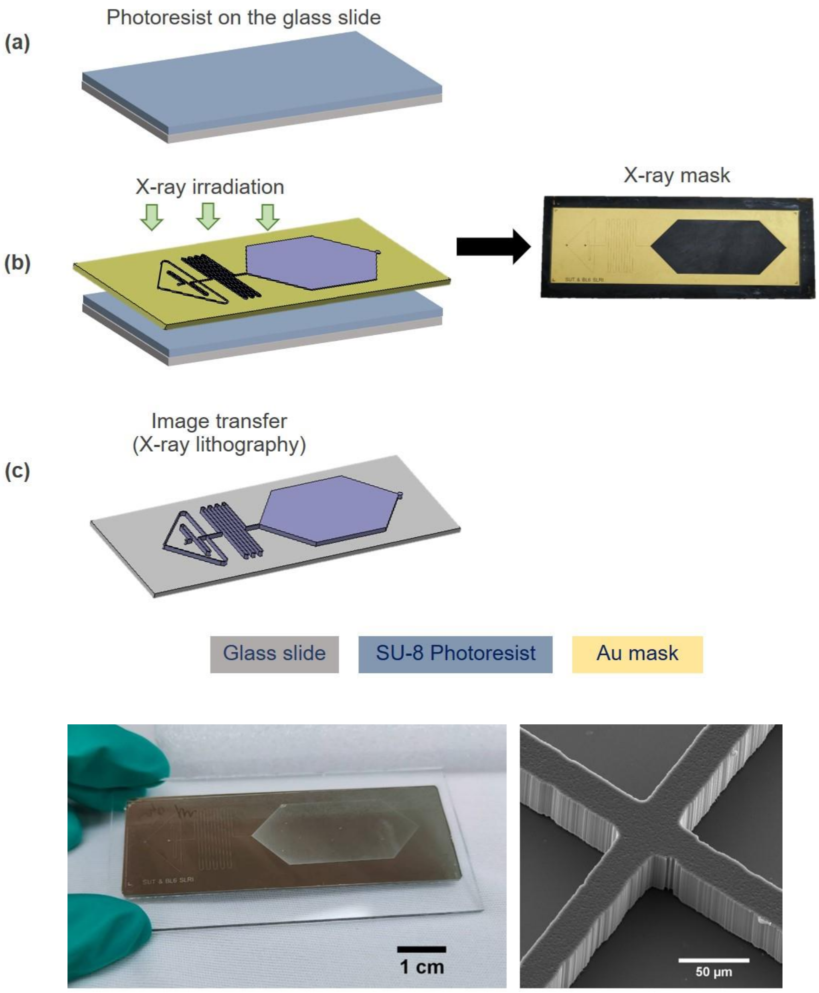

Figure S8. Fabrication of the SU-8 microchannel mold by using X-ray lithography. (a) The X-ray mask was placed on photoresist and set up in the X-ray scanning system of the BL6. (b) An X-ray exposure dose of 150,000 mJ cm^{-2} was applied, (c) SU-8 microchannel mold template for PDMS replication. The SU-8 microchannel mold template for PDMS replication has 50 µm microchannel height.

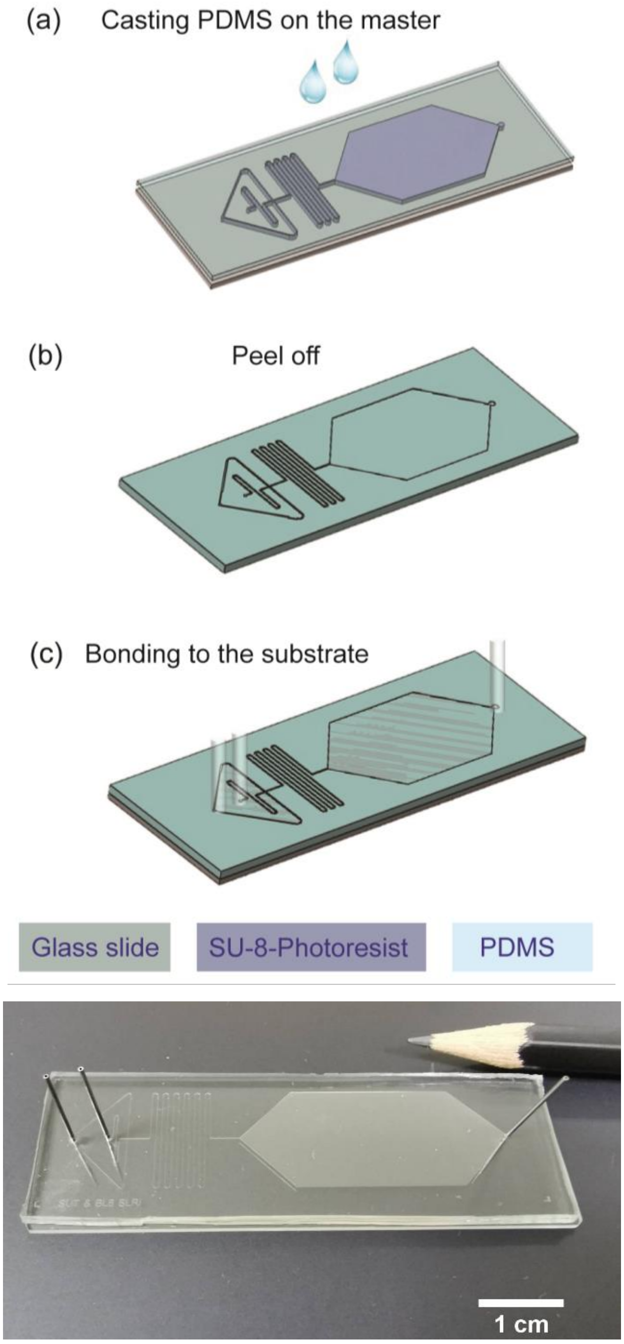

Figure S9. Fabrication of the microfluidic device by soft lithography. (a) The PDMS was poured and cross-linked over the SU- 8 master mold structure to fabricate the replica. (b) The PDMS was peeled off from the master to obtain the replica of the microfluidic chip. (c) The PDMS was permanently bonded to a glass surface by using oxygen plasma bonding and connected with silicone tubing.

Table S2. Comparison of the peak current, peak-to-peak separation (potential difference between oxidation peak potential, E_{ox}, and reduction peak potential, E_{red}), and electroactive surface area of the electrode with different modifications. The charge-transfer resistance and double-layer capacitance were estimated by fitting the equivalent circuits indicated in Figure 5. (n=3)

Electrode	Peak current (μA)	Peak-to-peak current separation (V)	Electroactive surface area (cm^2)	Charge-transfer resistance (Ω)		Double-layer capacitance (μF)	
				R_A	R_B	C_A	C_B
SPE	15.8 ± 0.6	0.239 ± 0.006	0.0148 ± 0.0004	3,240 ± 60	-	1.1 ± 0.1	-
Ppy (30 μm chip)/SPE	26.0 ± 1.0	0.195 ± 0.003	0.0224 ± 0.0007	120 ± 5.5	1501 ± 66	7.2 ± 0.9	30.3 ± 1.5
Ppy (10 μm chip)/SPE	32.3 ± 0.5	0.146 ± 0.001	0.0303 ± 0.0004	52.4 ± 1.9	545 ± 14	24.7 ± 0.8	96.2 ± 0.8

Table S3. Detection of glucose in synthetic urine (Sigma-Aldrich Sigmatrix) using the proposed GDH/poly-TBO/Ppy (10 μm chip)/SPE (n=3).

Glucose spike-in (mM)	Glucose measured (mM)	Recovery (%)	RSD (%)
0.500	0.475	95.1	3.9
1.00	0.969	96.9	1.9
